# Pro-angiogenic scaffold-free Bio three-dimensional conduit developed from human induced pluripotent stem cell-derived mesenchymal stem cells promotes peripheral nerve regeneration

**DOI:** 10.1038/s41598-020-68745-1

**Published:** 2020-07-21

**Authors:** Sadaki Mitsuzawa, Chengzhu Zhao, Ryosuke Ikeguchi, Tomoki Aoyama, Daisuke Kamiya, Maki Ando, Hisataka Takeuchi, Shizuka Akieda, Koichi Nakayama, Shuichi Matsuda, Makoto Ikeya

**Affiliations:** 1 Department of Orthopaedic Surgery, Kyoto University Graduate School of Medicine, 54 Shogoin Kawahara-cho, Sakyo-ku, Kyoto, 606-8507 Japan; 2 Department of Clinical Application, Center for iPS Cell Research and Application, Kyoto University, 53 Kawahara-cho, Shogoin, Sakyo-ku, Kyoto, 606-8507 Japan; 3 Department of Physical Therapy, Human Health Sciences, Kyoto University Graduate School of Medicine, Kyoto, Japan; 4 Takeda-CiRA Joint Program for iPS Cell Applications (T-CiRA), Fujisawa, Kanagawa Japan; 5 Cyfuse Biomedical K.K., Tokyo, Japan; 6 Department of Regenerative Medicine and Biomedical Engineering Faculty of Medicine, Saga University, Saga, Japan

**Keywords:** Neurogenesis, Peripheral nervous system, Regeneration and repair in the nervous system, Mesenchymal stem cells, Induced pluripotent stem cells

## Abstract

Although autologous nerve grafting is widely accepted as the gold standard treatment for segmental nerve defects, harvesting autologous nerves is highly invasive and leads to functional loss of the ablated part. In response, artificial nerve conduits made of artificial materials have been reported, but the efficacy of the nerve regeneration still needs improvement. The purpose of this study is to investigate the efficacy and mechanism of the Bio three-dimensional (3D) conduit composed of xeno-free human induced pluripotent stem cell–derived mesenchymal stem cells (iMSCs). The 5-mm nerve gap of the sciatic nerve in immunodeficient rats was bridged with the Bio 3D conduit or silicone tube. Functional and histological recovery were assessed at 8 weeks after surgery. The regenerated nerve in the Bio 3D group was significantly superior to that in the silicone group based on morphology, kinematics, electrophysiology, and wet muscle weight. Gene expression analyses demonstrated neurotrophic and angiogenic factors. Macroscopic observation revealed neovascularization both inside and on the surface of the Bio 3D conduit. Upon their subcutaneous implantation, iMSCs could induce angiogenesis. The Bio 3D conduit fabricated from iMSCs are an effective strategy for nerve regeneration in animal model. This technology will be useful in future clinical situations.

## Introduction

Peripheral nerve injury is often accompanied by trauma or tumor resection. The gold standard treatment when tensionless direct repair cannot be achieved is autologous nerve grafting^1,2^. Unlike solid organ transplantation such as heart, liver, kidney, or lung, a grafted nerve does not function itself, but offers the best scaffold for axonal elongation^3^. It does, however, have several potential disadvantages, including donor site morbidity, limited supply, risk of neuroma formation, mismatch of the caliber diameter, necessity of an extra surgical incision, and increased operative time^4^. A nerve allograft resolves some of these disadvantages, but requires perioperative immunosuppression and a particular preservation method^5^. Because peripheral nerve injury causes merely non-life threatening sensory and motor disturbance, the appropriateness of systemic immunosuppression therapy remains controversial.

An artificial nerve conduit represents a third option and is already used in selected clinical settings^6^. As the development of tissue engineering progresses, several studies have reported well-devised nerve conduits with improvements in the supportive cells, scaffolds, growth factors, and vascularity^7,8^. However, most of these conduits consist of artificial materials and are faced with the inevitable problems of low biocompatibility, foreign body reactions, and risk of infection.

To address these potential problems, we focused on novel technology of the Bio three-dimensional (3D) computer-controlled printing^9^. Nerve conduits created by Bio 3D printers are scaffold-free tubular tissues, composed entirely of homogenous multicellular spheroids without synthetic materials. Our previous study confirmed the efficacy of the Bio 3D conduit in peripheral nerve regeneration^10,11^. This concept of the Bio 3D conduit differs completely from others previously reported, and was considered to be a major breakthrough in the field of nerve regeneration. Originally, among all of the other cell types, primary dermal fibroblasts derived from adults were chosen to fabricate the spheroids for the Bio 3D conduits, because of their accessibility, proliferation, structural strength, and support for Schwann cells^10,12^. However, the quality of the fibroblasts depends on the donor’s state of health and is not easily stabilized, factors that could be major hurdles for off-the-shelf products.

Recent progress in induced pluripotent stem cell (iPSC)-based technology could provide a solution^13^. We have established a robust and simple protocol to induce mesenchymal stromal/stem cells (MSCs) from iPSCs through neural crest cell (NCC) lineage (iMSCs, hereafter)^14^. This stepwise induction protocol makes it possible to control and evaluate the quality and quantity of iMSCs. Moreover, compared to fibroblasts, MSCs are able to differentiate into a broad spectrum of end-stage cells, secrete a wide variety of immunomodulatory molecules, assemble exosomes, and repair damaged tissue via a mitochondrial transfer mechanism^15,16^. However, MSCs derived from bone marrow, adipose tissue, placenta, umbilical cord or other connective tissues have limitations, including accessibility, limited supply, invasive procedures, limited expandability and less differentiation potential during long-term culture. The quality of these MSCs also shows wide variation among donors. All of these problems could be resolved by iMSCs. iPSCs can ultimately be expanded^17^, and NCCs and iMSCs can be expanded over 10 passages^14,18^. Additionally, NCCs and iMSCs can be stored as frozen stocks. These characteristics offer great advantages for future clinical applications because, once NCCs were expanded and stored, over 10^10^ iMSCs could be obtained reproducibly from the same NCC stock, which will make it easier to check and control their quality. A neuroprotective effect of iMSCs is expected since peripheral glial cells are derived from NCCs. Further, NCC-derived endoneurial mesenchymal precursor cells have been shown to contribute to tissue repair and regeneration^19^, suggesting their regenerative potential for damaged tissues.

Thus, the purpose of the present study is to evaluate the Bio 3D conduit composed of human iMSCs in peripheral nerve regeneration in a rat sciatic nerve defect model. Our results indicate that the engrafted iMSCs accelerate nerve regeneration in terms of supportive cells, growth factors, and especially vascularity.

## Results

### Generating the Bio 3D conduit

**Figure 1:**
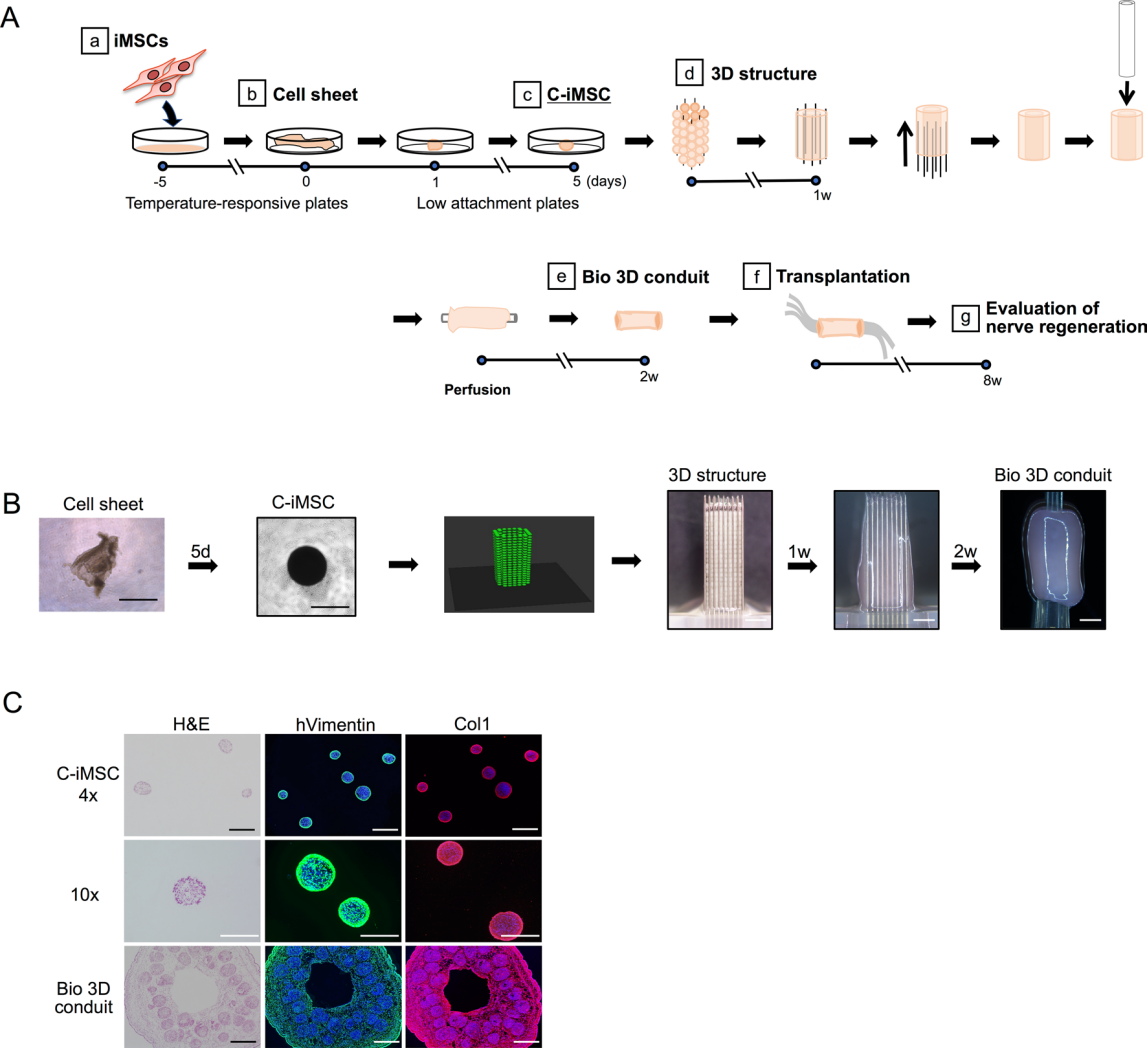
Generation of the Bio 3D conduit from xeno-free iMSCs. (**A**) Experimental design for the Bio 3D conduit. iMSCs are cultured on a temperature-responsive plate to generate a cell sheet. The cell sheets are then transferred to a low attachment plate to generate clumps of the iMSC/ECM complex, C-iMSCs. C-iMSCs are arranged into a needle array to generate the Bio 3D conduits and transplanted into a model rat. Eight weeks later, the recovery of the nerve injury was analyzed. (**B**) Images of cell sheets, C-iMSCs, pre-designed computer models, and Bio 3D structures in the Bio 3D conduit-generating step. After 1 week of perfusion, C-iMSCs are fused together and transferred to the catheter. Because the structures are shortened on the catheter, two conduits are fused together to achieve sufficient length. Scale bars: Cell sheet and C-iMSC, 500 µm; 3D structure and Bio-3D conduit, 2 mm. (**C**) Section images of C-iMSCs and the Bio 3D conduit. Staining with H&E (left) or anti-hVimentin (green, middle) and anti-Collagen1 (Col1) (red, right). Nuclei were stained with DAPI (blue). Scale bars, 500 µm.

In the following experiments, we used iMSCs induced from human iPSCs (hiPSCs) of a neural crest lineage under xeno-free conditions (see “Materials and methods”). iMSCs were cultured on temperature-responsive plates to generate cell sheets, and the cell sheets were then transferred to low-adhesion plates to generated clumps of iMSCs (C-iMSCs) (Fig. 1A,B)^20^. C-iMSCs with diameters of 500 ± 50 µm were arranged into a 9 × 9 needle array of a Bio-3D printer according to a pre-designed computer mode and then cultured with perfusion until adjacent C-iMSCs fused together. The fused conduits were then transferred onto a catheter and further cultured until the nerve conduit achieved a sufficient degree of strength. H&E and anti-human vimentin staining demonstrated the existence of iMSCs and the surrounding self-produced ECM complex in C-iMSCs and the Bio 3D conduits (Fig. 1C).

**Figure 2:**
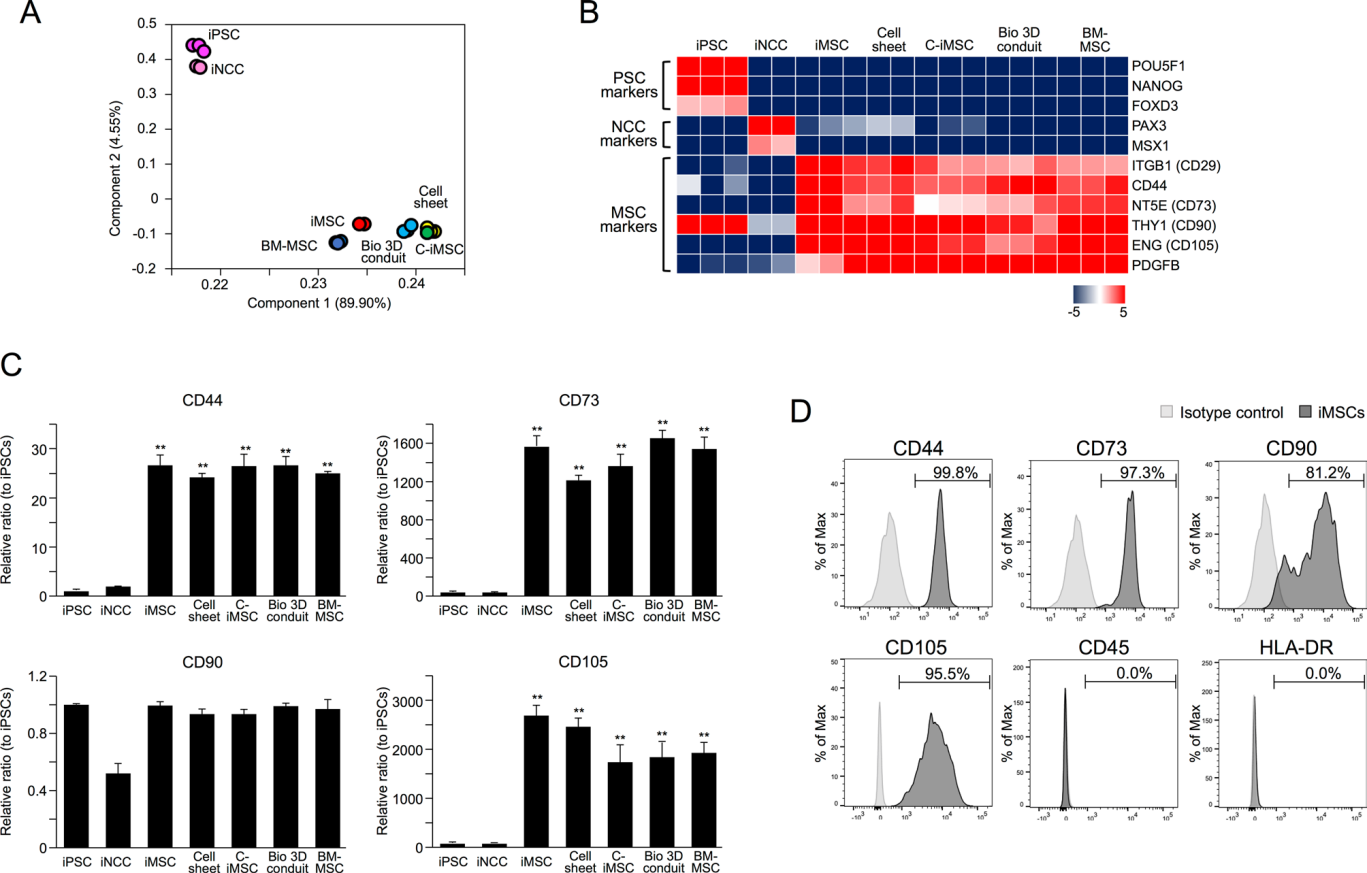
Cells composing the Bio 3D conduit maintained MSC properties. (**A**) Global gene expression profile. PCA analysis indicates no obvious change in the cell character in each Bio 3D conduit-generating step. (**B**) Heatmap illustrating the expression of pluripotent stem cell (PSC), neural crest cell (NCC), and mesenchymal stem cell (MSC) markers for cells in each Bio 3D conduit-generating step. (**C**) The mRNA expression of MSC marker genes was confirmed by RT-qPCR. MSC marker genes were expressed normally, indicating the MSC character was maintained. *P < 0.05, **P < 0.01 by Dunnett’s multiple comparisons *t* test compared with iPSCs. (**D**) iMSCs expressed surface markers for MSCs (CD44, CD90, CD90 and CD105), and were negative for CD45 and HLA-DR.

To investigate the properties of cells constituting nerve conduits, global gene expression profiles at each Bio 3D conduit-generating step were analyzed. Principal component analysis (PCA) and a heatmap of pluripotent stem cell (PSC), NCC, and MSC gene expressions revealed that the gene expression patterns of iMSCs, cell sheets, C-iMSCs, and the Bio 3D conduits were similar to bone marrow mesenchymal stem cells (BM-MSCs), and distinct of iPSC and iPSC-derived NCCs (iNCCs) (Fig. 2A,B). Additionally, the MSC markers such as CD44, CD73, CD90, and CD105 were normally expressed at each step (Fig. 2B,C). The successful induction of iMSCs was also confirmed by showing the characteristics of surface markers for MSC (positive for CD73, CD105, and CD44, and negative for CD45 and HLA-DR) (Fig. 2D).

### Transplantation and macroscopic observation of the Bio 3D conduit

**Figure 3:**
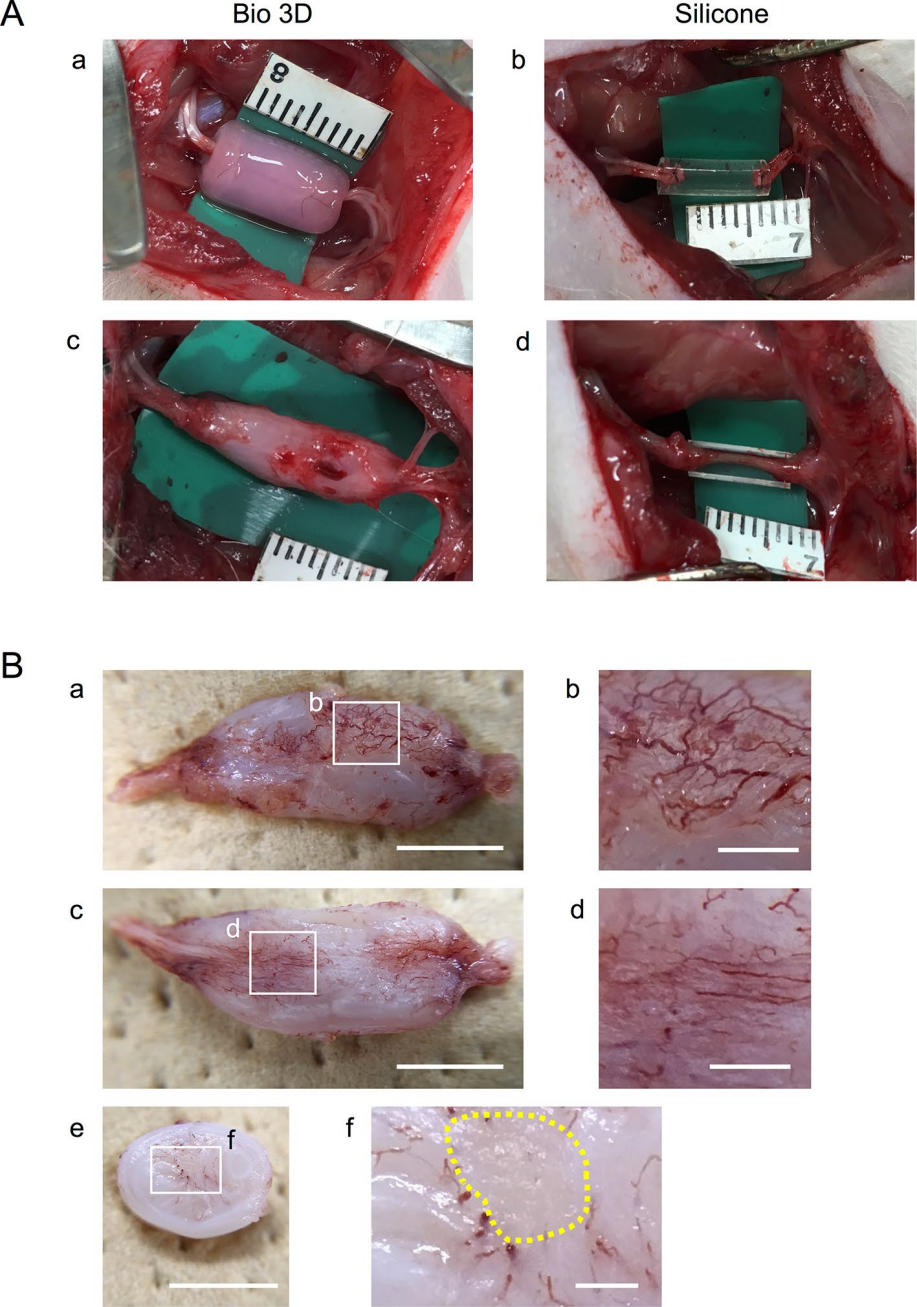
Transplantation of the Bio 3D conduit and macroscopic observation 8 weeks after surgery. (**A**) An 8-mm Bio 3D conduit (a) or silicone tube (b) was interposed into the sciatic nerve defect. The proximal and distal nerve stumps were pulled 1.5 mm into the conduit to create a 5-mm interstump gap. (c) Regenerated nerve 8 weeks after surgery. The engrafted Bio 3D conduit was not yet degraded, and its diameter remained larger than the proximal and distal stumps. (d) In the silicone group, although the nerve gap was successfully bridged, the regenerated nerve was very thin in the silicone tube. (**B**) Macroscopic observation of the harvested Bio 3D conduit 8 weeks after surgery. (a) Outer surface of the Bio 3D conduit shows newly formed blood vessels outside the conduit. An extended image of the white line box is shown as (b). (c) Longitudinally cut surface of the Bio 3D conduit shows newly formed blood vessels inside the conduit. An extended image of the white box is shown as (d). (e) A transversely cut surface revealed the newly formed blood vessels inside the conduit. An extended image of the white box is shown as (f). (f) A regenerated nerve at the center of the Bio 3D conduit (yellow dotted circle), and neovascularization around the regenerated nerve were confirmed. Scale bars: (a, c, e) 5 mm, (b, d, f) 1 mm.

All the Bio 3D conduits met the five criteria for the desired function and strength (retaining luminal structure, being easily graspable with forceps, having elastic force, being suitable for needle insertion, and being able to tolerate suturing), which is described in our previous study^11^. To examine the effect of the Bio 3D conduit, transplantation to the rat sciatic nerve defect model was performed (Fig. 3A-a,b). Eight weeks after the transplantation surgery, macroscopic observation was performed to check the morphology of the conduit and their effect on circumferential tissue (Fig. 3A-c,d). In both the Bio 3D conduit and silicone tube groups, the nerve gap was successfully bridged in all rats. In terms of degradation, the Bio 3D conduit maintained its shape macroscopically. Intriguingly, neovascularization was markedly observed at the superficial layer of the Bio 3D conduit (Fig. 3B-a,b). Transverse and longitudinal sections in the Bio 3D conduit showed a regenerated nerve at the center of the Bio 3D conduit, and neovascularization around the regenerated nerve inside the Bio 3D conduit (Fig. 3B-c–e, and yellow dotted circle in Fig. 3B-f). In contrast, in the silicone group, only a very thin regenerated nerve was observed in the silicone tube (Fig. 3A-d).

### Functional recovery of the regenerated nerve

To examine the functional recovery of the Bio 3D group, several evaluations were performed.

**Figure 4:**
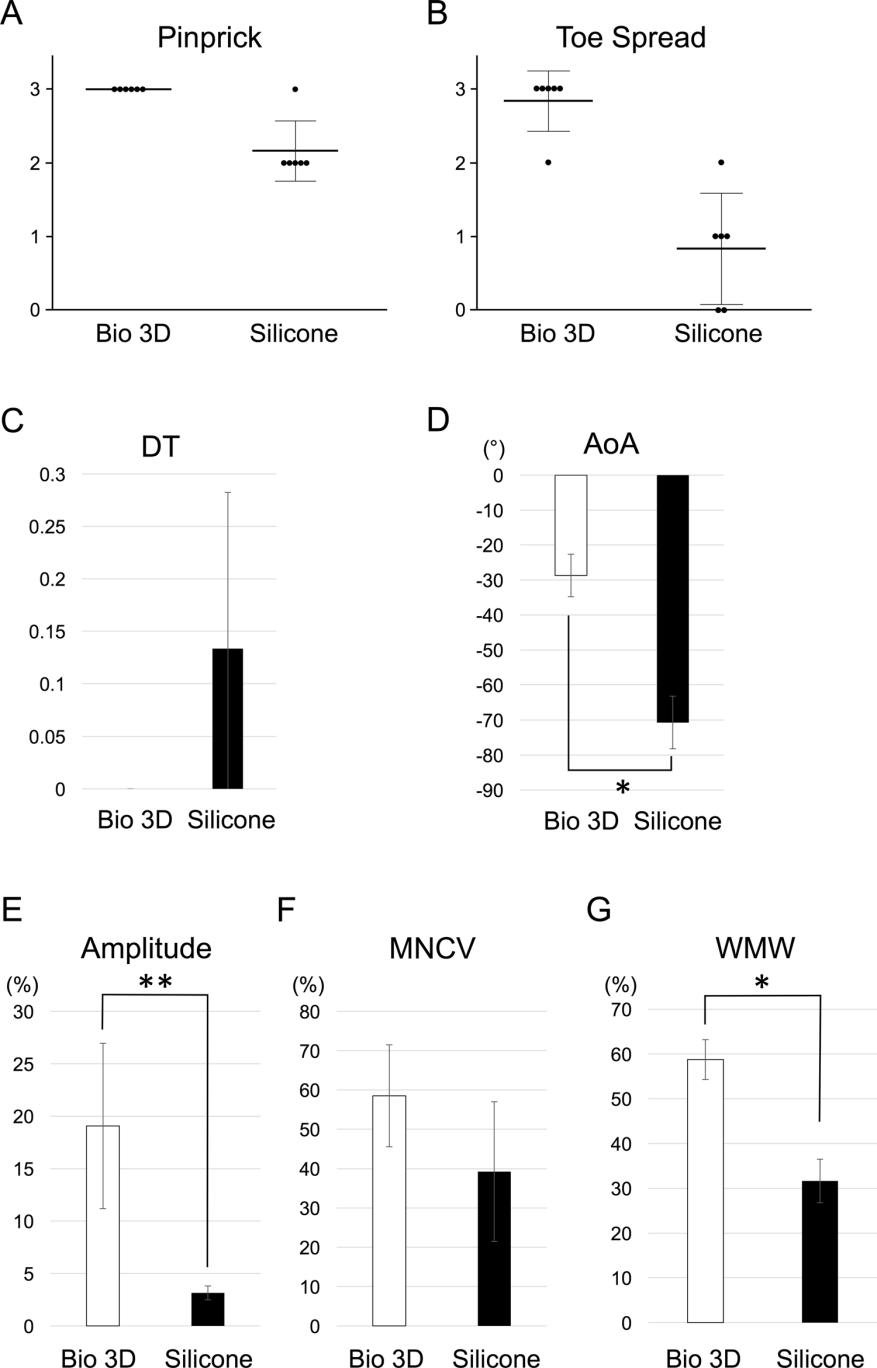
Functional recovery 8 weeks after surgery. (**A**) The pinprick test was performed to evaluate sensory recovery. All rats in the Bio 3D group were scored as grade 3. In the silicone group, one rat was scored as grade 3 and five rats were scored as grade 2. Although both groups showed functional recovery for the sensory nerve, there was a significant difference between the two groups (*P* < 0.01). (**B**) The toe-spread test was performed to evaluate motor recovery. In the Bio 3D group, five rats were scored as grade 3 and one rat was scored as grade 2. In the silicone group, one rat was scored as grade 2, three rats were scored as grade 1, and two rats were scored as grade 0. There was a significant difference between the two groups (*P* < 0.01). The Bio 3D group exhibited more functional recovery for the motor nerve. (**C**, **D**) Kinematic analysis showed that no drag toe (DT) was observed in the Bio 3D group. The Bio 3D group showed a significantly greater angle of attack (AoA) compared to the silicone group (*P < 0.01). (**E**, **F**) Electrophysiological study revealed significantly higher amplitude in the Bio 3D group (**P < 0.05). Motor nerve conduction velocity (MNCV) tended to be faster in the Bio 3D group, however, there was no significant difference between the two groups. (**G**) Wet muscle weight (WMW) of the tibialis anterior muscle. Little muscle atrophy was observed in the Bio 3D group with significance (*P < 0.01).

The pinprick test was performed to evaluate sensory recovery. Eight weeks after surgery, all rats in the Bio 3D group were scored as grade 3 (Fig. 4A). In the silicone group, one rat was scored as grade 3 and five rats were scored as grade 2. Although both groups showed functional recovery for the sensory nerve, there was a significant difference between the two groups (*P* < 0.01), indicating the Bio 3D group exhibited more functional recovery of the sensory nerve.

The toe-spread test was performed to evaluate motor recovery. Eight weeks after the surgery, in the Bio 3D group, five rats were scored as grade 3 and one rat was scored as grade 2 (Fig. 4B). In the silicone group, one rat was scored as grade 2, three rats were scored as grade 1, and two rats were scored as grade 0. There was a significant difference between the two groups (*P* < 0.01), indicating the Bio 3D group exhibited more functional recovery of the motor nerve.

To evaluate behavioral functional recovery, the kinematic properties of the hind limbs were measured while the rat was walking on a treadmill. The mean drag toe (DT) was 0.0 ± 0.0 in the Bio 3D group and 0.13 ± 0.15 in the silicone group (Fig. 4C). There was no significant difference between the two groups. The Bio 3D group showed a significantly greater angle of attack (AoA) (− 28.70° ± 6.08°) than the silicone group (− 70.72° ± 7.49°) (*P* < 0.01) (Fig. 4D). According to Tajino et al., the AoA of the intact nerve group and the non-treatment (just dissected) group were approximately − 1° and − 47°, respectively^21^. The silicone group in this study showed worse function than the non-treatment group in the literature, which is because the follow up time of 8 weeks aggravated the function of the extensor digitorum muscle to extend the toes until the regenerated nerve bridged between the stumps. Although the Bio 3D group in this study showed improved function for walking behavior compared to the silicone group, it is still inferior to the intact nerve in the previous study.

To confirm that the newly elongated axon will reach the neuromuscular junction of the target muscle, we performed an electrophysiological study of the pedal adductor muscle. The Bio 3D group exhibited a significantly greater amplitude than the silicone group (19.07 ± 7.88% vs. 3.15 ± 0.66%, respectively; *P* < 0.05) (Fig. 4E). The mean motor nerve conduction velocity (MNCV) was higher in the Bio 3D group than in the silicone group (58.52 ± 12.96% vs. 39.23 ± 17.77%, respectively), although the difference was not significant (Fig. 4F). These data indicated that the target muscle was more strongly re-innervated in the Bio 3D group. According to Zhang et al., the amplitude values of the nerve autograft group, the Bio 3D (human-gingiva derived MSCs) group, and the silicone group were approximately 65%, 52%, and 28%, respectively^22^. Although our results were not as good as in their report, the different study protocol (rat facial nerve, 5-mm gap, 12 weeks follow up) might explain this. Once a nerve has begun to regenerate through the Bio 3D conduit (human iMSCs), increasing compound muscle action potentials (CMAPs) can be expected over time.

To check for muscle atrophy caused by peripheral nerve injury, the wet muscle weight of the tibialis anterior muscle was measured. The wet muscle weight was significantly heavier in the Bio 3D group than the silicone group (58.77 ± 4.44% vs. 31.65 ± 4.87%, respectively; *P* < 0.01) (Fig. 4G), indicating target muscle atrophy in the silicone group was greater than in the Bio 3D group. According to Santiago et al. and Sowa et al. (rat sciatic nerve, 5- or 6-mm gap, 12 weeks follow up), the wet muscle weights of the gastrocnemius muscle in the nerve autograft group, the collagen tube group, and the non-treatment group were 66.6%, approximately 40%, and 19.2%, respectively^23,24^. In the non-treatment group, no regeneration was observed between proximal and distal nerve stumps. Because the wet muscle weight of the tibialis anterior muscle in the current silicone group was 31.65%, we can compare the Bio 3D group (58.77%) to those in previous studies above to some extent. Little muscle atrophy in the Bio 3D group suggested that the target muscle was re-innervated from the early stage of recovery.

### Histological examination of the regenerated nerve

To evaluate the quantity and quality of the regenerated nerve, histological and morphometric analyses were performed.

**Figure 5:**
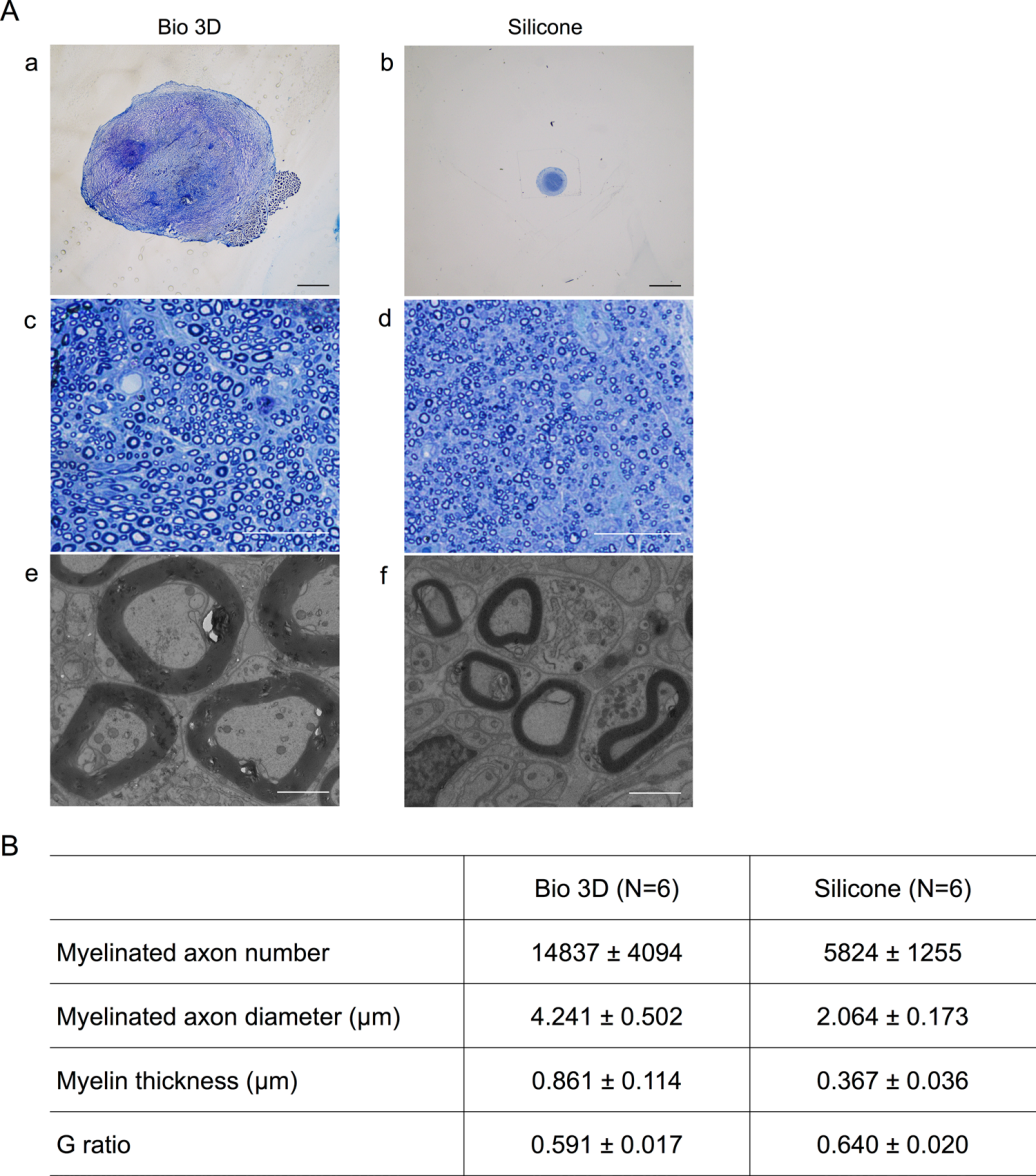
Histological and morphometric evaluation of the regenerated nerve. (**A**) (a–d) Semi-thin transverse sections (toluidine blue staining) under light microscopy. (e, f) Ultra-thin transverse sections under transmission electron microscopy. The mid-portion of the Bio 3D group exhibited an obviously larger cross-sectional area than the silicone group did. The regenerated nerve of the Bio 3D group also exhibited many well myelinated axons. Scale bars: (a, b) 1,000 μm, (c, d) 50 μm, (e, f) 2 μm. (**B**) The Bio 3D group showed greater myelinated axon number, larger axon diameter, and thicker myelin formation. The G ratio of the Bio 3D group was smaller than that of the silicone group. All data showed significant differences between the two groups (*P* < 0.01).

Semi-thin toluidine blue staining of the mid-portion in the Bio 3D group exhibited many well myelinated axons (Fig. 5A-a,c,e) compared to the silicone group (Fig. 5A-b,d,f). The Bio 3D group showed a significantly larger myelinated axon number (14,837 ± 4,094) than the silicone group (5,824 ± 1,255) (*P* < 0.01) (Fig. 5B). Transmission electron microscopy (TEM) revealed myelinated axons with proper myelin sheaths existed in the Bio 3D group. The Bio 3D group also showed a significantly larger myelinated axon diameter (4.241 ± 0.502 μm) compared to the silicone group (2.064 ± 0.173 μm) (*P* < 0.01) and significantly thicker myelin formation (0.861 ± 0.114 μm vs. 0.367 ± 0.036 μm) (*P* < 0.01). Finally, the G ratio of the Bio 3D group was significantly smaller (0.591 ± 0.017) than the silicone group (0.640 ± 0.020) (*P* < 0.01). Overall, the morphology of the regenerated axons was demonstrated to be superior to those in the silicone group. According to Yurie et al. and Yamamoto et al., the total number of myelinated axons of the intact nerve group, the nerve autograft group, the Bio 3D (human fibroblast) group, and the collagen conduit group were 11,351, 14,345, 6,516, and 5,417, respectively^10,25^. Because of the short follow up time of 5 weeks in the study design of Yamamoto et al., the larger number for the nerve autograft group might indicate axonal sprouting from the proximal nerve stump. The Bio 3D (human iMSCs) group in the current study also demonstrated a similar result. As to the quality of the newly elongated axons, Yurie et al. reported that the myelinated axon diameter/myelin thickness values of the intact nerve group were 4.122 μm/1.112 μm, and those of the Bio 3D (human fibroblast) group were 2.189 μm/0.623 μm. The regenerated nerves of the Bio 3D (human iMSCs) group in this study and the intact nerve group in the previous study were very alike in terms of axonal quality. In summary, the regenerated nerve through the current Bio 3D conduit (human iMSCs) was superior in terms of both the quantity and quality of the myelinated axons.

### Pro-neurogenic and pro-angiogenic potential of the Bio 3D conduit

**Figure 6:**
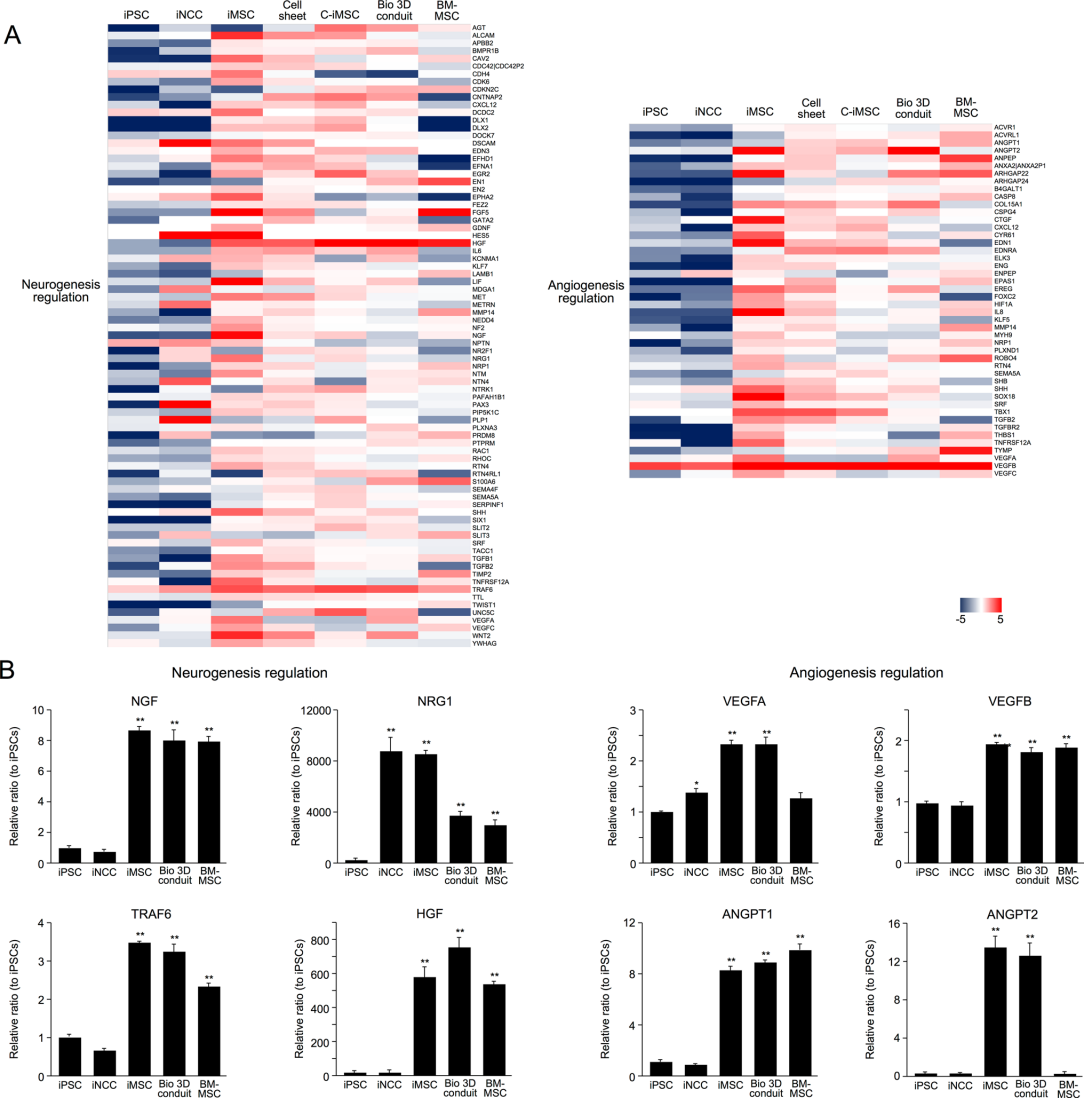
The Bio 3D conduit promotes neurogenesis and angiogenesis. (**A**) Heatmap illustrating the expression of neurogenesis and angiogenesis related genes in iPSCs, iNCCs, cells in each Bio 3D conduit-generating step, and BM-MSCs. By convention, highly expressed genes are shown in red and lowly expressed genes are shown in blue. iMSCs in each Bio 3D conduit-generating step included a large subset of upregulated neurogenesis (left) and angiogenesis (right) related genes. Gene names and values used to obtain the heatmap are shown in Table S2. (**B**) The mRNA expression of neurogenesis and angiogenesis genes was confirmed by RT-qPCR. **P < 0.01, by Dunnett’s multiple comparisons *t* test compared with iPSCs.

The benefits of iMSCs on tissue regeneration have been associated with the ability to produce a broad variety of cytokines and paracrine factors^20^. Therefore, we evaluated whether the Bio 3D conduit expressed pro-neurogenic and pro-angiogenic factors. Global gene expression profiles revealed iMSCs in each Bio 3D conduit-generating step including a large subset of upregulated neurogenesis and angiogenesis related genes in the Gene Ontology (GO) categories (Fig. 6A, Table S2).

RT-qPCR confirmed similar patterns of representative neurogenesis (NGF, NRG1, TRAF6 and HGF)- and angiogenesis (VEGFA, VEGFB, ANGPT1, and ANGPT2)-related gene expressions (Fig. 6B). These data suggest that the Bio 3D conduits (pre-transplantation) have the potential to support nerve regeneration through their pro-neurogenic and pro-angiogenic capability.

### Angiogenetic potential of iMSCs

**Figure 7:**
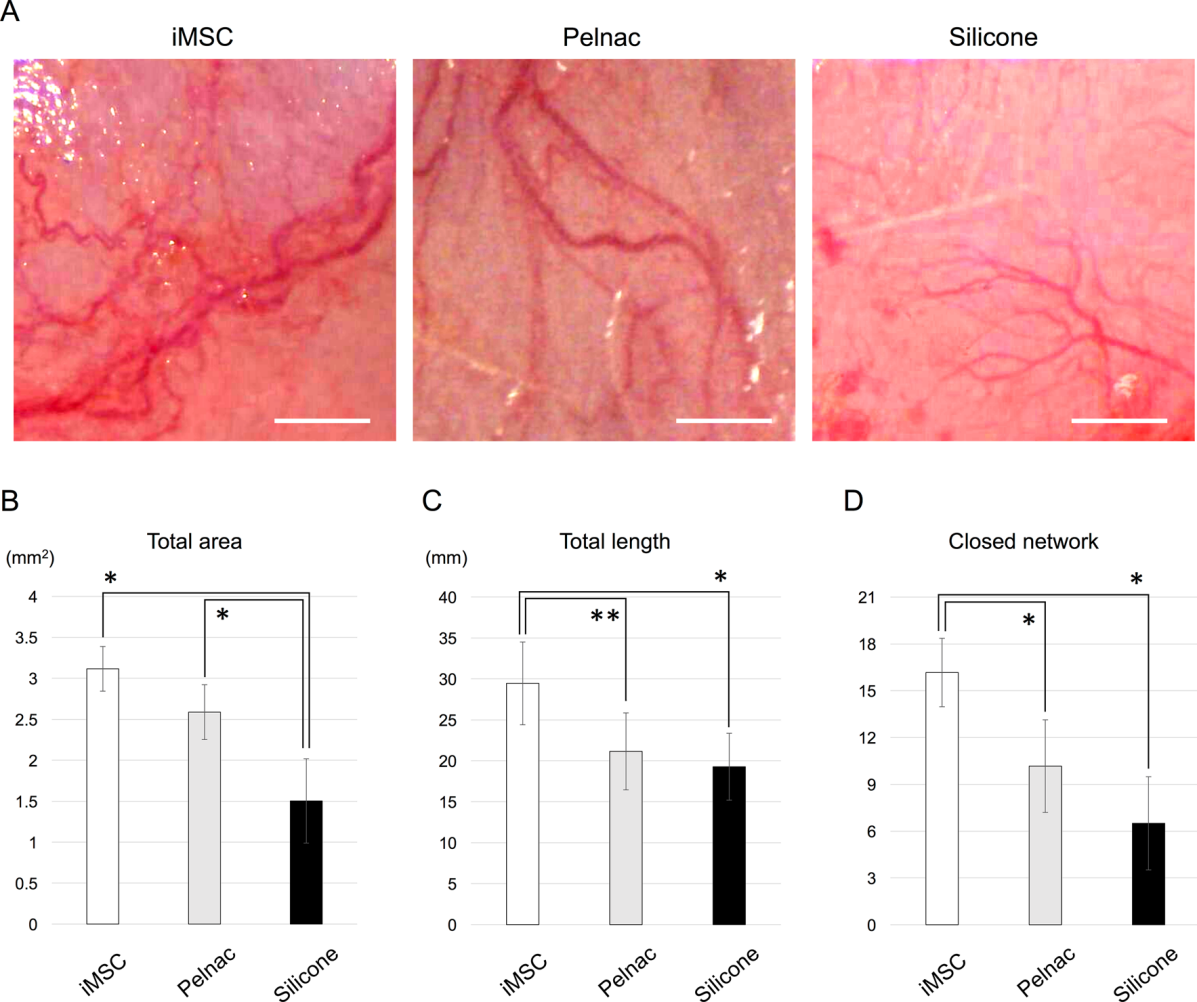
Subcutaneous implantation for the angiogenesis assay. (**A**) Gross photos under an operative microscope in three groups. The iMSC group exhibited a greater amount of neovascularization. Scale bars, 1 mm. (**B**) The total area of angiogenesis of the iMSC group tended to be larger than that of the Pelnac group and was significantly larger than that of the silicone group (*P < 0.01). (**C**) The total length of angiogenesis of the iMSC group was significantly longer than that of the Pelnac group (**P < 0.05) and the silicone group (*P < 0.01). (**D**) The number of closed networks of angiogenesis of the iMSC group was significantly larger than that of the other two groups (*P < 0.01).

Since pro-angiogenic factors were expressed in the Bio 3D conduit and abundant neovascularization around the Bio 3D conduit was observed macroscopically (Fig. 3B), the angiogenetic property of iMSCs was tested by transplanting iMSCs under the rat dorsal skin soaked with an atelocollagen sponge (Pelnac; Gunze, Kyoto, Japan). Gross photos under an operative microscope at 2 weeks after transplantation are shown in Fig. 7A. The total area of angiogenesis of the iMSC group was 3.12 ± 0.27 mm^2^, which tended to be larger than that of the Pelnac group (2.59 ± 0.33 mm^2^) and was significantly larger than that of the silicone group (1.50 ± 0.51 mm^2^, *P* < 0.01) (Fig. 7B). Consistently, the total length of angiogenesis of the iMSC group was 29.45 ± 5.05 mm, which was significantly longer than that of the Pelnac group (21.15 ± 4.70 mm, *P* < 0.05) and the silicone group (19.27 ± 4.08 mm, *P* < 0.01) (Fig. 7C). Finally, the number of closed networks of angiogenesis of the iMSC group was 16.17 ± 2.19, which was significantly larger than that of the Pelnac group (10.17 ± 2.97, *P* < 0.01) and the silicone group (6.50 ± 2.99, *P* < 0.01) (Fig. 7D). These results revealed that subcutaneously transplanted iMSCs induce newly formed vessels more strongly than Pelnac alone as the control group (the Pelnac group).

## Discussion

The present study demonstrated the efficacy and mechanism of the Bio 3D conduit developed from iMSCs on peripheral nerve regeneration. Although the effectiveness of the Bio 3D conduit for peripheral nerve regeneration was confirmed in previous reports in the case of fibroblasts^10,11^ and of gingiva-derived MSCs^22^, the present study is novel in that we used iPSC-derived MSCs for the first time. The cell type is a key issue of the regenerative medicine for future clinical application of neurological disorders.

Several studies have reported that the essential components for nerve regeneration include scaffolds^26,27^, vascularity^28–30^ supportive cells^23,25^, and growth factors^31,32^. In terms of scaffolds, the Bio 3D conduit in the present study was composed of pure biological tissues fabricated from homogenous cells. Eliminating synthetic materials diminishes the risk of foreign-body reactions, infections, and allergies. Although the Bio 3D conduit does not offer the same microstructure as a scaffold, its wall is sufficiently thick and substantial to retain its lumen, hold anchoring sutures, and resist surrounding scar formation. From the standpoint of vascularity, preinserted vessels inside artificial nerve conduits promote capillary formation and are useful for nerve regeneration^28–30^. The Bio 3D conduit in this study adopted a different method from that above. iMSCs should acquire additional viability through perfusion cultivation before transplantation and induce larger amounts of circumferential blood supply in the living body compared to artificial nerve conduits. In the present study, the engrafted Bio 3D conduit was less likely to become necrotic or degrade 8 weeks after surgery based on macroscopic observation. We investigated newly formed blood vessels macroscopically both at the superficial and deep layers of the Bio 3D conduit and found the new vessels to have mainly originated from blood vessels accompanying the original sciatic nerve stumps (Fig. 3B). An angiogenesis assay confirmed the capacity of iMSCs to induce neovascularity, which is consistent with the VEGF expression in the Bio 3D conduit. From the perspective of supportive cells, MSCs and other cell types transplanted into the chamber of a conduit have been shown to differentiate into Schwann-like cells and to support axonal elongation^24,33,34^. However, leakage of the implanted cells from the chamber or the necessity of cell migration to artificial material is a matter of issue ^35,36^. Instead, the Bio 3D conduit in the current study can bear far more cells unless it has collapsed, thus its seeding efficacy is obviously superior to that of other methods reported so far. In other studies, various growth factors were added to the nerve conduit by direct injection into the lumen or other delivery systems^31,32^. Due to a loss of quantity through leakage or short-term retention, these efforts are often less effective. The Bio 3D conduit could solve these concerns if fabricated from genetically manipulated cells to overexpress neurotrophic factors^37^. This is a point for improvement in future studies.

Although several Bio 3D printing technologies for tubular constructions have been reported, we chose to use the recently developed Kenzan method^9,10,38^. Using this novel technology with a simple computer system, researchers can freely design the construction in terms of form, diameter, and length. Scaffold-free tubular tissue have already been applied to blood vessels and tracheal cartilage^9,39^. In the field of peripheral nerve regeneration, the efficacy of the Bio 3D conduit has already been investigated, but there is still room for improvement regarding constituent cell types^10,11,22^. Looking ahead to future clinical applications, we selected here iMSCs to generate Bio 3D conduits.

A previous study confirmed that the newly formed blood vessels within the nerve gap play a key role in providing Schwann cells with a scaffold for crossing the nerve gap and offering a more conductive environment for axonal regrowth^40^. Therefore, vascularity is one of the essential components of nerve regeneration, and macroscopic observation in the current study confirmed the formation of newly formed blood vessels both inside and outside of the Bio 3D conduit (Fig. 3B). In further detail, there is a marked neovascularization at the superficial layer of the Bio 3D conduit. This may be explained by the difference in the blood supply. Namely, blood flow of the deep layer of the Bio 3D conduit is served only by the blood vessels that accompany the sciatic nerve. In addition to this, the superficial layer of the Bio 3D conduit is supplied with further blood flow by the surrounding soft tissue (in this case, the gluteus maximus muscle). Additional experiments also support the capacity of iMSCs to induce angiogenesis (Fig. 7). Taken together, utilization of the Bio 3D conduit generated by our method may be advantageous for nerve regeneration therapy.

There are several limitations associated with the present study. First, we did not perform a control study with a nerve autograft or other commercial synthetic conduit. Because autologous nerve grafting is considered the gold standard treatment, further improvements to the efficacy of the Bio 3D conduit are essential before comparison. Future research of other cell types to constitute the Bio 3D conduit, co-culture of several types of cells to create spheroids, and overexpression of beneficial cytokine with genetic manipulation could help the Bio 3D conduit catch up with nerve autografting^41^. As far as was possible, the Bio 3D conduit in the current study was compared to other treatment options from previous studies, including the Bio 3D conduit fabricated from other types of cells (human fibroblast or human gingiva-derived MSCs)^10,21–25^. Second, our experimental design involved a fairly small interstump gap of 5 mm, which is not critical in rats and also not long enough to assess nerve regeneration. However, this is the first study to use iMSCs to fabricate the Bio 3D conduit, and we regard the finding of trustworthy nerve regeneration over a short gap as more important than a less reliable outcome over a longer and more challenging gap. Although a longer-length Bio 3D conduit would be time-and-cost consuming and technically demanding, future study should confirm the efficacy of the Bio 3D conduit over longer gaps.

We confirmed that the Bio 3D conduit composed of feeder-free and xeno-free human iMSCs contributes to peripheral nerve regeneration. Although further research on safety, effectiveness, and cost should be performed before clinical application, the Bio 3D conduit should become a useful alternative to nerve autografts in the treatment of nerve defects.

## Materials and methods

### iMSC culture

Human iPSCs (1231A3) were cultured on an iMatrix-511 (Nippi, Tokyo, Japan)-coated cell culture plate in StemFit AK03N (Ajinomoto, Tokyo, Japan) as described previously^42^. The xeno-free method to induce human iMSCs from human iPSCs is described elsewhere [D. Kamiya, personal communication]. Briefly, Cells were cultured in Stemfit Basic03 (Ajinomoto), which is comparable to AK03N minus FGF2, supplemented with SB431542 (Sigma, St. Louis, MO, USA) and CHIR99021 (Wako, Osaka, Japan), to induce NCCs. CD271^+^ cells were then sorted by FACS and expanded onto fibronectin-coated plates in Stemfit Basic03 supplemented with SB431542, EGF (R&D, Minneapolis, USA), and FGF2. iMSCs were induced and maintained with PRIME-XV MSC Expansion XSFM medium (Fujifilm Irvine Scientific, Tokyo, Japan). The cells were diluted at a ratio of 1:3 when they reached 70–80% confluence. Cells at passage 3 were used for the experiments. Human bone marrow mesenchymal stem cell (hBM-MSC) was obtained from PromoCell (Heidelberg, Germany) and cultured in PRIME-XV MSC Expansion XSFM medium.

### Preparation of C-iMSCs

Clumps of an iMSC/extracellular matrix (ECM) complex (C-iMSCs) were prepared as reported previously with minor modifications^20,43^. Briefly, iMSCs were seeded at a density of 5 × 10^4^ cells/well in temperature-responsive 96-well plates (UpCell, CellSeed, Inc., Tokyo, Japan) coated with fibronectin and cultured with Prime-XV MSC expansion XSFM for 4 days. To obtain C-iMSCs, confluent cells that had formed on the cellular sheet with iMSC-derived ECM were detached from the dishes by switching the culture temperature from 37 to 27 °C. The iMSC/ECM complexes were detached from the bottom of the plate in a sheet shape, transferred to a 96-well low-cell-adhesion plate (Sumitomo Bakelite, Tokyo, Japan), and rolled up to generate a round clump of cells. The cell clumps were maintained in Prime-XV MSC expansion XSFM for 5 days.

### Preparation of the Bio 3D conduit

To assemble the conduits, C-iMSCs were robotically placed into skewers of a 9 × 9 needle array using the “Kenzan method”; C-iMSCs were arranged in a three-dimensional shape according to a pre-designed 3D model by the Bio-3D printer^10^. The C-iMSCs were then cultured in a perfusion bioreactor to promote self-organization of the living cells. Approximately 1 week after 3D printing, adjacent C-iMSCs were fused to construct a single tubular shape, removed from the Kenzan array, transferred to a perforated 18-gauge intravenous catheter (2 cm) (Surflo; Nipro, Osaka, Japan), and further cultured for 2 weeks until the desired length (8 mm) and strength was achieved.

### Immunocytochemistry

Paraffin-embedded sections were deparaffinized, and antigen retrieval was performed by microwave (300 W, 20 min). Samples were blocked with Blocking One (Nacalai Tesque, Inc.) for 1 h. DAPI (10 μg/mL) was used to counterstain nuclei. Samples were observed by BZ-9000E (Keyence, Osaka, Japan). The antibodies used in this study are summarized in Table S3.

### Quantitative PCR analysis

Quantitative PCR analysis was performed as described previously^44^. Briefly, Total RNA was purified with an RNeasy Kit (Qiagen, Valencia, CA, USA) and treated with a DNase-one Kit (Qiagen, MD, USA) to remove genomic DNA. Total RNA (0.1 µg) was reverse transcribed for single-stranded cDNA using random hexamers and Superscript III reverse transcriptase (Invitrogen, CA, USA) according to the manufacturer’s instructions. Quantitative PCR was performed with the Thunderbird SYBR qPCR Mix (Toyobo, Osaka, Japan) and analyzed with the StepOne real-time PCR system (Applied Biosystems, Waltham, MA, USA). Primer sequences are listed in Table S1.

### Fluorescence-activated cell sorting

Fluorescence-activated cell sorting (FACS) was performed by AriaII (BD) according to the manufacturer’s protocol. Antibodies used in FACS are listed in Table S3.

### Transcriptome analysis

Total RNA was purified using an RNeasy Micro Kit (Qiagen) and treated with the DNase-one Kit (Qiagen) to remove genomic DNA. Briefly, 10 ng of total RNA was transcribed to obtain single-stranded cDNA using the SuperScript VILO cDNA Synthesis Kit (Thermo Fisher Scientific). A cDNA library was synthesized using the Ion AmpliSeq Transcriptome Human Gene Expression Core Panel (Thermo Fisher Scientific) and Ion Ampliseq Library Kit Plus (Thermo Fisher Scientific) according to the manufacturer’s protocol. Barcode-labeled cDNA libraries were analyzed by the Ion S5 XL System (Thermo Fisher Scientific) using the Ion 540 Chip Kit (Thermo Fisher Scientific). Total RNA was purified using the RNeasy Micro Kit (Qiagen) and treated with the DNase-one Kit (Qiagen) to remove genomic DNA. We reverse transcribed 10 ng of total RNA to obtain single-stranded cDNA using the SuperScript VILO cDNA Synthesis Kit (Thermo Fisher Scientific). We performed cDNA library synthesis using the Ion AmpliSeq Transcriptome Human Gene Expression Core Panel (Thermo Fisher Scientific) and the Ion Ampliseq Library Kit Plus (Thermo Fisher Scientific) according to the manufacturer’s protocol. Barcode-labeled cDNA libraries were analyzed by the Ion S5 XL System (Thermo Fisher Scientific) using the Ion 540 Chip Kit (Thermo Fisher Scientific).

### In vivo experimental design

All animal procedures were performed with approval of and in accordance with guidelines of the ethics committee of Kyoto University Graduate School of Medicine, Animal Experimentation Committee. Fifteen adult male F344-rnu/rnu rats with immune deficiency (7-weeks old, weighing 120–160 g) (Clea, Tokyo, Japan) were used in this study. Rats were randomly divided into Bio 3D (n = 6) and silicone (n = 6) groups for the nerve regeneration study. The remaining three rats were prepared for the additional experiments as part of the angiogenesis assay. Each rat was acclimatized before the surgical procedures, housed in a separate cage, and given standard food and water.

### Surgical technique

Under general anesthesia, a longitudinal skin incision was made at the posterior thigh in the prone position. The right sciatic nerve was exposed through a gluteal muscle splitting approach and sharply cut at the middle of the thigh to create a nerve defect. In the Bio 3D group, an 8-mm Bio 3D conduit was interposed between the proximal and distal stumps. Under a surgical microscope, each stump was then pulled 1.5 mm into the conduit and anchored in place with epineural 9-0 nylon sutures, creating a 5-mm interstump gap in the conduit. The wound was closed in layers with 5-0 nylon sutures. In the silicone group, the silicone tube (8 mm in length and 2 mm in internal diameter) was interposed in the same procedure. All of the following analyses and sample harvesting were performed 8 weeks after surgery.

### Pinprick test

A pinching stimulus was applied by means of standardized forceps from the toe to the heel until the rat withdrew. Each animal’s response to the pinprick test was graded from 0 to 3 as follows: grade 0, no response to stimulus; grade 1, withdrawal response at the heel; grade 2, withdrawal response on the dorsum of the foot; grade 3, withdrawal response at the toes^45^.

### Toe-spread test

Intact rats extend and abduct their toes when suspended by the base of the tail. The degree of toe movement was graded as follows: grade 0, no movement; grade 1, any sign of movement; grade 2, toe abduction; grade 3, toe abduction with extension^45^.

### Kinematic analysis

Before the walking analysis, rats were anesthetized, and six landmarks for each hind limb were marked as previously described^10,21,46^. Briefly, colored hemispheric plastic markers were bilaterally attached onto the shaved skin at five landmarks: the anterior superior iliac spine, hip joint, knee joint, lateral malleolus, and fifth metatarsophalangeal joint. To detect the toes, acrylic resin ink was applied to the tip of the bilateral middle toe. Hind limb motion was captured at a sampling rate of 120 Hz using a 3D motion capture apparatus (Kinema Tracer System; Kissei Comtec, Nagano, Japan) while rats walked at a pace of 10 cm/s. For each rat, a total of five or six consecutive steps was included in the following analysis. Markers were traced, and 3D displacements were reconstructed by the system. Two parameters were calculated in this study: (1) DT, which measures the proportion of the step to the total analyzed step in which the rat’s toe was grounded during the swing phase, and (2) AoA, which measures the toe angle to the metatarsal bone at the final segment of the swing phase. A smaller DT value represents less limping. A larger AoA value indicates more dorsal flexion in the toe just before the rat’s foot is grounded.

### Electrophysiological studies

Electrophysiological studies about the sciatic nerve was performed as described previously^10,30^. Briefly, the bilateral sciatic nerves were exposed at the prone position under general anesthesia. The right sciatic nerve was stimulated just distal to the piriformis muscle (S1) and at the popliteal fossa (S2) by means of an electromyogram measuring system (Neuropack S1; Nihon Kohden, Tokyo, Japan). Two pairs of needle electrodes were inserted into the pedal adductor muscle to check for the CMAPs. The amplitude (peak to peak) of the CMAPs evoked with supramaximal electric stimulation from S1 was measured. The MNCV was calculated from the distance between S1 and S2. The same procedure was performed on the left intact hind limb. The amplitude and MNCV of the CMAPs in the pedal adductor muscle of the right hind limb were expressed as a percentage of those in the left hind limb.

### Wet muscle weight of the tibialis anterior muscle

After harvesting the regenerated nerve, the bilateral tibialis anterior muscles were exposed and detached from the bone at their origin and insertion, and weighed immediately using a digital scale, as described previously^10,30^.

### Histological and morphometric studies

The regenerated nerve was harvested from six rats in each group, fixed in 1% glutaraldehyde and 1.44% paraformaldehyde, post-fixed with 1% osmic acid, and embedded in epoxy resin. Transverse sections (1 μm in thickness) of the mid-portion of the regenerated nerves were prepared and stained with 0.5% (w/v) toluidine blue solution and examined by light microscopy (Nikon Eclipse 80i; Nikon, Tokyo, Japan). Ultra-thin sections of the same tissues stained with uranyl acetate and lead citrate were evaluated using TEM (Model H-7000; Hitachi High-Technologies, Tokyo, Japan). Total myelinated axon number, the myelinated axon diameter, myelin thickness, and the G-ratio were measured using ImageJ software (National Institute of Health, USA) for morphometric analysis as described previously^10,28–30^. Briefly, the total neural area (a) of each specimen was calculated by choosing six or seven fields at random so that the area analyzed would represent > 20% of the entire neural area of each specimen. The number of myelinated axons (b), neural area (c), shortest diameter of each myelinated axon (d), and axon diameter (e) were calculated for each field at a final magnification of 400 ×. The number of myelinated axons and neural areas from all analyzed fields were summed. The total number of myelinated axons in each specimen was estimated as b × (a/c). The mean myelinated axon diameter was expressed as the average value of the shortest diameter of all myelinated axons, the mean myelin thickness was expressed as (d − e)/2, and the G-ratio was expressed as e/d using the mean value in the seven fields evaluated.

### Subcutaneous implantation for angiogenesis assay

To examine the angiogenesis potential of iMSCs, three rats were used to compare the iMSC, Pelnac (Pelnac; Gunze, Kyoto, Japan), and silicone. Under general anesthesia, six separate 1-cm transverse incisions at 2-cm intervals were made on the backs of the rats^47,48^. In the iMSC group, six square atelocollagen sponges (4 × 4 × 2 mm in size, Pelnac) were soaked with 20 μL of complete medium of iMSCs and implanted into each incision under the dorsal skin. In the Pelnac group, six square atelocollagen sponges (4 × 4 × 2 mm in size, Pelnac) with 20 μL of complete medium only were implanted. In the silicone group, six square silicone sheets (4 × 4 × 2 mm in size) were implanted. Two weeks after the implantation, a midline incision on the backs was made to observe newly formed blood vessels on the sponges and silicone sheets, and gross photos were taken under an operative microscope. The total area, total length, and the number of closed networks were analyzed using ImageJ software (National Institute of Health, USA) for newly developed angiogenesis as described in previous studies^49,50^.

### Statistical analyses

Data are presented as the means and standard deviations. Data analyses of the pinprick test and toe spread test were performed with the Wilcoxon test in JMP (JMP Pro 14.0; SAS Institute, Cary, NC, USA). Data analyses of the kinematic analysis, the electrophysiological studies, wet muscle weight of the tibialis anterior muscle, and the histological and morphometric studies were performed using Student’s *t* test in Microsoft Excel 2017 (Microsoft, Redmond, WA, USA). Values of *P* < 0.05 were considered statistically significant. Data of the angiogenesis assay were compared using one-way analysis of variance (ANOVA). When a significant difference was detected, a post hoc test was applied using the Tukey Kramer test (JMP Pro 14.0). Values of *P* < 0.05 were considered statistically significant.

## Supplementary information

Supplementary Information 1.

Supplementary Information 2.

Supplementary Information 3.

Supplementary Information 4.

## Data Availability

The data that support the findings of this study are available from the corresponding author upon reasonable request.
