# Supplementary material for: Pro-angiogenic scaffold-free Bio three-dimensional conduit developed from human induced pluripotent stem cell-derived mesenchymal stem cells promotes peripheral nerve regeneration
Source: Sci Rep. 2020 Jul 21;10:12034. doi: 10.1038/s41598-020-68745-1 (PMC7374629; doi:10.1038/s41598-020-68745-1)
Supplement: Supplementary file 1 — Supplementary Information 1. [file 41598_2020_68745_MOESM1_ESM.docx]

Pro-angiogenic scaffold-free Bio three-dimensional conduit developed from human induced pluripotent stem cell–derived mesenchymal stem cells promotes peripheral nerve regeneration

Sadaki Mitsuzawa^1^ *, Chengzhu Zhao^2^*, Ryosuke Ikeguchi^1^**, Tomoki Aoyama^3^, Daisuke Kamiya^2,4^, Maki Ando^1^, Hisataka Takeuchi^1^, Shizuka Akieda^5^, Koichi Nakayama^6^, Shuichi Matsuda^1^, Makoto Ikeya^2,4^**

1 Department of Orthopaedic Surgery, Kyoto University Graduate School of Medicine, Kyoto, Japan

2 Department of Clinical Application, Center for iPS Cell Research and Application, Kyoto University, Kyoto, Japan

3 Department of Physical Therapy, Human Health Sciences, Kyoto University Graduate School of Medicine, Kyoto, Japan

4. Takeda-CiRA Joint Program for iPS Cell Applications (T-CiRA), Fujisawa, Kanagawa, Japan

5 Cyfuse Biomedical K.K., Tokyo, Japan

6 Department of Regenerative Medicine and Biomedical Engineering Faculty of Medicine, Saga University, Saga, Japan

* These authors contributed equally to this work.

** Address correspondence to

Ryosuke Ikeguchi, Department of Orthopaedic Surgery, Kyoto University Graduate School of Medicine, 54 Shogoin Kawahara-cho, Sakyo-ku, Kyoto 606-8507, Japan. Tel: +81-75-751-3657; Fax: +81-75-751-8409; Email: ikeguchir@me.com

Makoto Ikeya, Department of Clinical Application, Center for iPS Cell Research and Application, Kyoto University, 53 Kawahara-cho, Shogoin, Sakyo-ku, Kyoto 606-8507, Japan. Tel: +81-75-366-7054; Fax: +81-75-366-7073; Email: [mikeya@cira.kyoto-u.ac.jp](mailto:mikeya@cira.kyoto-u.ac.jp)

Table S1. qPCR Primers List

Table S2. Values of genes of different cell types used to obtain heatmap (Related to Fig. 6)

Table S3. Antibodies List
